# Redox-sensitive iodinated polymersomes carrying histone deacetylase inhibitor as a dual-functional nano-radiosensitizer for enhanced radiotherapy of breast cancer

**DOI:** 10.1080/10717544.2021.1995080

**Published:** 2021-11-03

**Authors:** Zhehong Zhu, Manran Wu, Juan Sun, Zhengyuan Huangfu, Lingling Yin, Weipeng Yong, Jing Sun, Guanglin Wang, Fenghua Meng, Zhiyuan Zhong

**Affiliations:** a Biomedical Polymers Laboratory, College of Chemistry, Chemical Engineering and Materials Science, State Key Laboratory of Radiation Medicine and Protection, Soochow University, Suzhou, China; b State Key Laboratory of Radiation Medicine and Protection, School of Radiation Medicine and Protection, School for Radiological and Interdisciplinary Sciences (RAD-X), Collaborative Innovation Center of Radiation Medicine of Jiangsu Higher Education Institutions, Soochow University, Suzhou, China

**Keywords:** Radiotherapy, polymersomes, SAHA, drug delivery, SPECT

## Abstract

Radiotherapy (RT) is a frequently used means in clinical tumor treatment. The outcome of RT varies, however, to a great extent, due to RT resistance or intolerable dose, which might be resolved by the development of radio-sensitizing strategies. Here, we report redox-sensitive iodinated polymersomes (RIP) carrying histone deacetylase inhibitor, suberoylanilide hydroxamic acid (SAHA, vorinostat), as a new dual-functional nano-radiosensitizer for breast cancer radiotherapy. SAHA-loaded RIP (RIP-SAHA) with a size of about 101 nm exhibited good colloidal stability while the reduction-activated release of SAHA, giving rise to better antitumor effect to 4T1 breast carcinoma cells than free SAHA. Accordingly, RIP-SAHA combined with a 4 Gy dose of X-ray radiation led to significantly enhanced suppression of 4T1 cells compared with SAHA combined 4 Gy of X-ray radiation, as a result of enhanced DNA damage and impeded DNA damage repair. The pharmacokinetics and biodistribution studies by single-photon emission computed tomography (SPECT) with ^125^I-labeled SAHA (^125^I-SAHA) showed a 17.3-fold longer circulation and 237.7-fold better tumor accumulation of RIP-SAHA over SAHA. The systemic administration of RIP-SAHA greatly sensitized radiotherapy of subcutaneous 4T1 breast tumors and brought about significant inhibition of tumor growth, without causing damages to major organs, compared with radiotherapy alone. RIP not only enhanced SAHA delivery but also acted as a radiosensitizer. RIP-SAHA emerges as a smart dual-functional nano-radiosensitizer to effectively enhance tumor radiotherapy.

## Introduction

Radiotherapy (RT) is one of the most widely used and effective treatments for malignant solid tumors in clinical settings (Citrin, 2017; Pallares & Abergel, 2020; Lehrer et al., 2021). The anticancer effect of RT is based on DNA damage of cancer cells *via* high-energy photons or charged particles (Meidanchi et al., 2015; Jiang et al., 2018a, 2018b; Hahn et al., 2021). The outcome of RT varies, however, to a great extent, because some tumor cells are resistant to RT and patients can’t tolerate high dose X-ray irradiation. In the past years, great interest lies in the development of safe radio-sensitizing strategies that can boost RT for malignant tumors without escalating X-ray irradiation dose (Song et al., 2017; Dong et al., 2020; Xia et al., 2020; Manoharan et al., 2021). There are two strategies used to improve the therapeutic efficiency of radiotherapy. The first one used materials containing high Z elements, such as gold nanoparticles, bismuth-based nanoparticles, hafnium-based nanoparticles, gadolinium-based nanoparticles, and platinum nanoparticles to enhance X-ray absorption, generate electrons and induce a large amount of free radicals (Liu et al., 2017; Yu et al., 2017; Li et al., 2019; Kuang et al., 2020; Wu et al., 2021). However, the clinical translation of inorganic materials is limited by their high cost and safety issues. Although iodinated organic nanoparticles, for instance, iodinated liposomes, nanoemulsions, dendritic polymers, and nanoparticles, display good biocompatibilities (Attia et al., 2014; Jin & Lu, 2014; Zou et al., 2018; Hainfeld et al., 2020), the X-ray absorption is inadequate due to the low iodine content. In previous work, we reported that iodinated polymersomes had an ultra-high iodine content, long blood circulation, and good tumor targeting ability (Zou et al., 2019), which might be of interest for enhanced radiotherapy.

Another important and widely used strategy is to increase DNA damage and prevent DNA damage repair by radiosensitizers like histone deacetylase (HDAC) inhibitors (Jiang et al., 2017; Cheng et al., 2019; Zong et al., 2019). HDAC plays an important role in the transcriptional regulation by stabilization of DNA histone interaction and deacetylation process (Wang et al., 2015; Luan et al., 2019; Ortega et al., 2019). HDAC inhibitors, e.g. suberoylanilide hydroxamic acid (SAHA, vorinostat) could decrease the interaction between histone and DNA, leading to enhanced exposure of DNA double strands and thus radiosensitivity to irradiation (Gerelchuluun et al., 2018; Liu et al., 2020). As the first FDA approved HDAC inhibitor, SAHA is used for the treatment of cutaneous T-cell lymphoma and undergoes clinical trials for breast cancer, small cell lung cancer, etc. (Grant et al., 2007; Sankar et al., 2015; Zeng et al., 2016; Rodriguez et al., 2020; Lu et al., 2021). The side effects of SAHA, such as allergic reaction, cardiac toxicity, and diarrhea, poor water solubility induced by the highly non-polar nature of SAHA, and poor tumor targeting ability, however, limit its clinical efficacy (Cai et al., 2010; Duvic & Dimopoulos, 2016; Kaur et al., 2021). Liposomes with prolonged circulation were shown to improve the delivery of SAHA into tumor (Xiao et al., 2019).

**Scheme 1.**
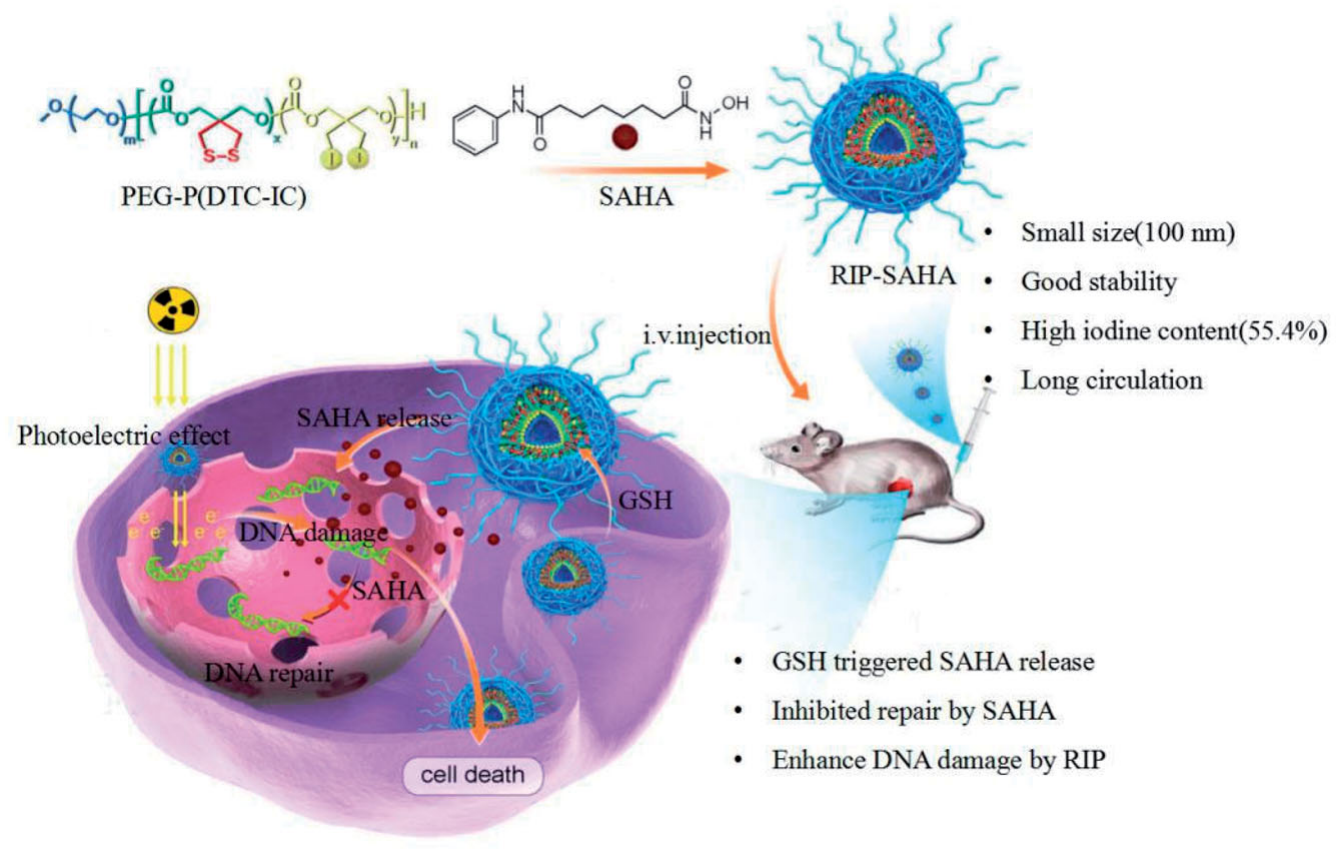
Schematic illustration of RIP-SAHA as a dual-functional nano-radiosensitizer for synergistically enhanced radiotherapy of breast cancer *via* enhancing DNA damage and impeding DNA damage repair.

In this study, we designed redox-sensitive iodinated polymersomes (RIP) carrying SAHA, as a dual-functional nano-radiosensitizer for enhanced breast cancer radiotherapy (Scheme 1). RIP is disulfide-crosslinked to improve its stability and accelerate intracellular drug release, as reported previously for delivery of different drugs (Liu et al., 2020; Fu et al., 2021; Gong et al., 2021). RIP not only possesses a high iodine content of 55.4 wt.% but also efficiently loads and delivers SAHA into tumor cells. These dual-functional nano-radiosensitizer has been shown to significantly improve tumor accumulation and tumor radiotherapy compared with RIP and SAHA alone.

## Experiment

### Preparation and characterization of RIP-SAHA

Poly(ethylene glycol)-b-poly(dithiolane trimethylene carbonate-co-iodinated trimethylene carbonate) (PEG-P(DTC-IC) triblock polymer [Mw: 5.0–(3.8–45.8) g/mol] was synthesized as previous reported (Zou et al., 2019). For ^125^I radiolabeling, 100 µL PEG-P(DTC-IC) in DMF was added into 200 µCi of ^125^I and 5 µL of ice acetic acid and reacted at 80 °C for 4 h. The radiolabeling efficiency was examined by thin-layer chromatography (TLC) using saline as a mobile phase. Rf of ^125^I-PEG-P(DTC-IC) ∼ 0.1, Rf of free ^125^I ∼ 0.9 was obtained by the same method. For ^125^I radiolabeling of SAHA (^125^I-SAHA), 100 µL SAHA dissolved in DMF (10 mg/mL) and added into 200 µCi of ^125^I solution in iodogen tube. The mixture reacted for 40 min at 37 °C. The radiolabeling efficiency was examined by TLC using saline as a mobile phase. Rf of ^125^I-SAHA ∼ 0.1, Rf of free ^125^I ∼ 0.9 was obtained by the same method.

To prepare RIP, 100 μL PEG-P(DTC-IC) in DMF (10 mg/mL) was dropped to 890 μL phosphate buffer solution (PBS) and waited for 15 min. The same method was used for the preparation of SAHA encapsulated RIP (RIP-SAHA) and ^125^I-labeled RIP (^125^I-RIP).

The hydrodynamic size and distribution of RIP and RIP-SAHA were measured by dynamic light scattering (DLS). All the tests were carried on a Malvern Zetasizer Nano ZS90 equipped with a solid-state He-Ne laser (*λ* = 633 nm) three times at 25 °C. To visualize the morphology, RIP (10 µL, 1 mg/mL) was dropped onto a carbon-coated copper grid of 200 mesh. After removing the redundant liquid, phosphotungstic acid aqueous solution (1 mg/mL, 10 µL) was dropped onto the copper grid. The sample was characterized by a transmission electron microscope (TEM, FEI Tecnai F20) at 120 kV.

### In vitro release of SAHA

To investigate the release of SAHA, ^125^I-labeled SAHA-loaded RIP (RIP-^125^I-SAHA) was added into a dialysis bag and against PBS with or without 10 mM DTT. The release of SAHA was measured at 1, 2, 4, 6, 8, 12, 24, 48, and 72 h by using a gamma counter. The same method was used for the releasing of SAHA after 4 Gy radiations radiation.

### Cytotoxicity assay

MTT assays were used to evaluate the cytotoxicity of RIP-SAHA with or without radiation. 4T1 breast cancer cells were seeded in a 96-well plate and incubated for 12 h. Then, the medium was extracted and the cell was washed with PBS three times. Following, SAHA, RIP, or RIP-SAHA was added into the cell and incubated for 24 h with or without 4 Gy of X-ray radiation at the concentration of SAHA from 1 to 500 µM. After 24 h, the cell was washed three times with PBS and added 100 µL of MTT solution to each well with another 4 h incubation. In the end, the MTT was washed and DMSO was added into each well. The absorbance of each cell was measured by a microplate reader (Thermo, Varioskan Flash).

### Immunofluorescence cell assay

4T1 cells seeded on the 24 cell plate were incubated with SAHA, RIP, or RIP-SAHA for 12 h, and then irradiated with 4 Gy of X-ray radiation. At 2 and 24 h post-irradiation, the cells were washed with PBS three times and treated with Triton X-100 (0.5%) for 15 min to rupture the cell membrane. The cell was washed with PBS another three times and added 500 µL of 5% bovine serum albumin (BSA) to each well for 1 h at 37 °C. Following, the cell was incubated with 100 µL of γ-H2AX antibody in the dark overnight at 4 °C, washed with PBS for three times, and incubated in rabbit anti-mouse secondary antibody for 1 h at 37 °C in the dark. In the end, the cell nuclei were, stained with DAPI (200 μL, 5 mg/mL) for 5 min. The fluorescence images were taken by confocal laser scanning microscope (Olympus FV1200) and cell damage was counted by using the ImageJ software.

### ROS detection

4T1 cells (5 × 10^4^) seeded on the 24 cell plate were incubated for 12 h. SAHA (500 µM), RIP (41.2 µM), or RIP-SAHA (SAHA: 500 µM) was added into the cells for another 12 h incubation. 4 Gy of X-ray irradiation was used to irradiate the cells. DCFH-DA (20 µM) was added into cells for 30 min incubation. Then, the cells were washed by PBS three times and fixed by paraform for 10 min. In the end, the cell nuclei were stained with DAPI (200 μL, 5 mg/mL) for 5 min. The fluorescence images were taken by confocal laser scanning microscope (Olympus FV1200) and cell damage was counted by using the ImageJ software.

### Pharmacokinetics and biodistribution

All animal studies were operated under the guideline of the Animal Care and Use Committee of Soochow University. Female BALB/c mice (18–20 g per mice, 6 weeks of age) of specific pathogen-free (SPF) grade were received from Shanghai SLAC Laboratory Animal Co., Ltd. To investigate the pharmacokinetics, ^125^I-SAHA (20 µCi) or SAHA loaded ^125^I-labeled RIP (^125^I-RIP-SAHA) (20 µCi) was intravenously injected into healthy mice. The blood was collected from the mouse orbit at a pre-determined time point and weighed. The radioactivity of blood was measured by a gamma counter.

For the biodistribution study, 4T1 breast cancer cell bearing (200 mm^3^) BALB/c mice were used by intravenous injection of ^125^I-SAHA (200 µCi) or ^125^I-RIP-SAHA (200 µCi). The *micro*SPECT/CT was scanned at 0, 4, 8, 12, 24, 48, and 72 h with SPECT scan was set at 15 min, and CT scan at 55 keV tube voltages, and 615 mA tube current. After imaging, the organs were harvested, weighed and the radioactivity was measured by a gamma counter.

### In vivo therapy

The 4T1 subcutaneous tumor (50 mm^3^) mice were randomly divided into seven groups: PBS, 4 Gy, SAHA, RIP-SAHA, SAHA + 4 Gy, RIP + 4 Gy, and RIP-SAHA + 4 Gy, with 6 mice in each group. SAHA, RIP, or RIP-SAHA (SAHA: 2 mg/Kg) was administered *via* the tail vein on days 0, 3, and 6, and X-rays (4 Gy) were irradiated to the tumor on days 2, 5, and 8, respectively. The tumor volume was measured with a vernier caliper and weighed with a balance every two days. Three days after the treatment, a mouse was randomly killed in each group, major organs and tumors were slices for H&E and TUNEL staining.

### Statistical analysis

One-way analysis of variance (ANOVA) was used to assess significance between groups, after which *post-hoc* tests with the Bonferroni correction were used for comparison among individual groups. *p < .05 was considered significant, **p < .01 and ***p < .001 considered highly significant.

## Results and discussion

### Preparation and characterization of RIP-SAHA

**Figure 1.**
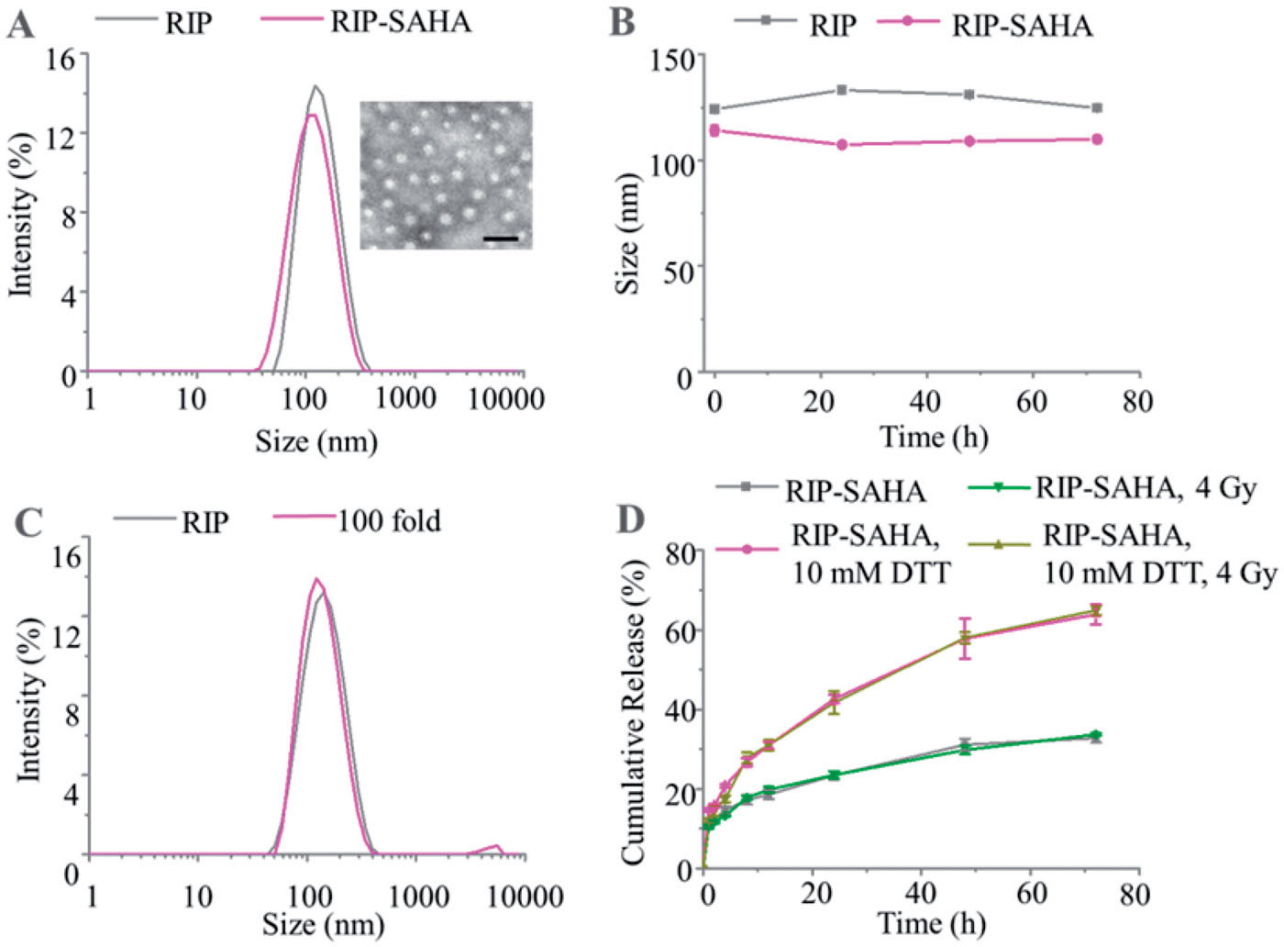
(A) Size distribution profile of RIP and RIP-SAHA and morphology of RIP measured by DLS and TEM (The scale bar corresponds to 200 nm). (B) Colloidal stability of RIP and RIP-SAHA measured by DLS over time. (C) Stability of 100 times dilution of RIP measured by DLS. (D) Cumulative release of SAHA in 10% FBS over time with or without 10 mM DTT and 4 Gy of X-ray radiation.

The iodine-rich copolymer, poly(ethylene glycol)-*b*-poly(dithiolane trimethylene carbonate-*co*-iodine trimethylene carbonate) [PEG-P(DTC-IC), *M_n_* = 5.0–(3.8–45.8) kg/mol], was synthesized by ring-open copolymerization of DTC and IC using MeO-PEG-OH as an initiator, as reported previously (Zou et al., 2019). To accurately study the pharmacokinetics and biodistribution, PEG-P(DTC-IC) was radiolabeled with ^125^I in DMF at 80 °C by isotope exchange method. The radiolabeling efficiency increased with the extension of reaction time, achieving a plateau efficiency of 80% at 4 h, as revealed by thin-layer chromatography (TLC) (Figure S1). To investigate *in vivo* behavior, SAHA was radiolabeled with ^125^I by oxidant method with iodogen as an oxidizing agent in DMF at room temperature. TLC showed a radiolabeling efficiency of 42.1% (Figure S2). RIP and RIP-SAHA were prepared by solvent exchange method in the absence and presence of SAHA, respectively. The dynamic light scattering (DLS) showed a hydrodynamic size of 125 nm with narrow distribution for RIP (Figure 1(A)). Transmission electron microscopy (TEM) confirmed that RIP had a vesicular structure. RIP showed an efficient loading of SAHA, achieving drug loading efficiency (DLE) of 70.5 ± 1.9% and drug loading content (DLC) of 6.4 ± 0.2% under theoretical loading content of 9.1%. The colloidal stability studies displayed that RIP and RIP-SAHA were stable against PBS and 10% fetal bovine serum (FBS) over 72 h (Figure 1(B)). Further, DLS measurements showed that RIP was stable with extensive dilution (100-fold) in PBS (Figure 1(C)). To simulate the redox conditions *in vivo*, dithiothreitol (DTT) was used to study the stability of RIP. In the presence of DTT, however, RIP became unstable and displayed two peaks (Figure S3), supporting its redox-sensitivity as reported for disulfide-crosslinked systems (Chen et al., 2017; Yang et al., 2018; Gao et al., 2020).

**Figure 2.**
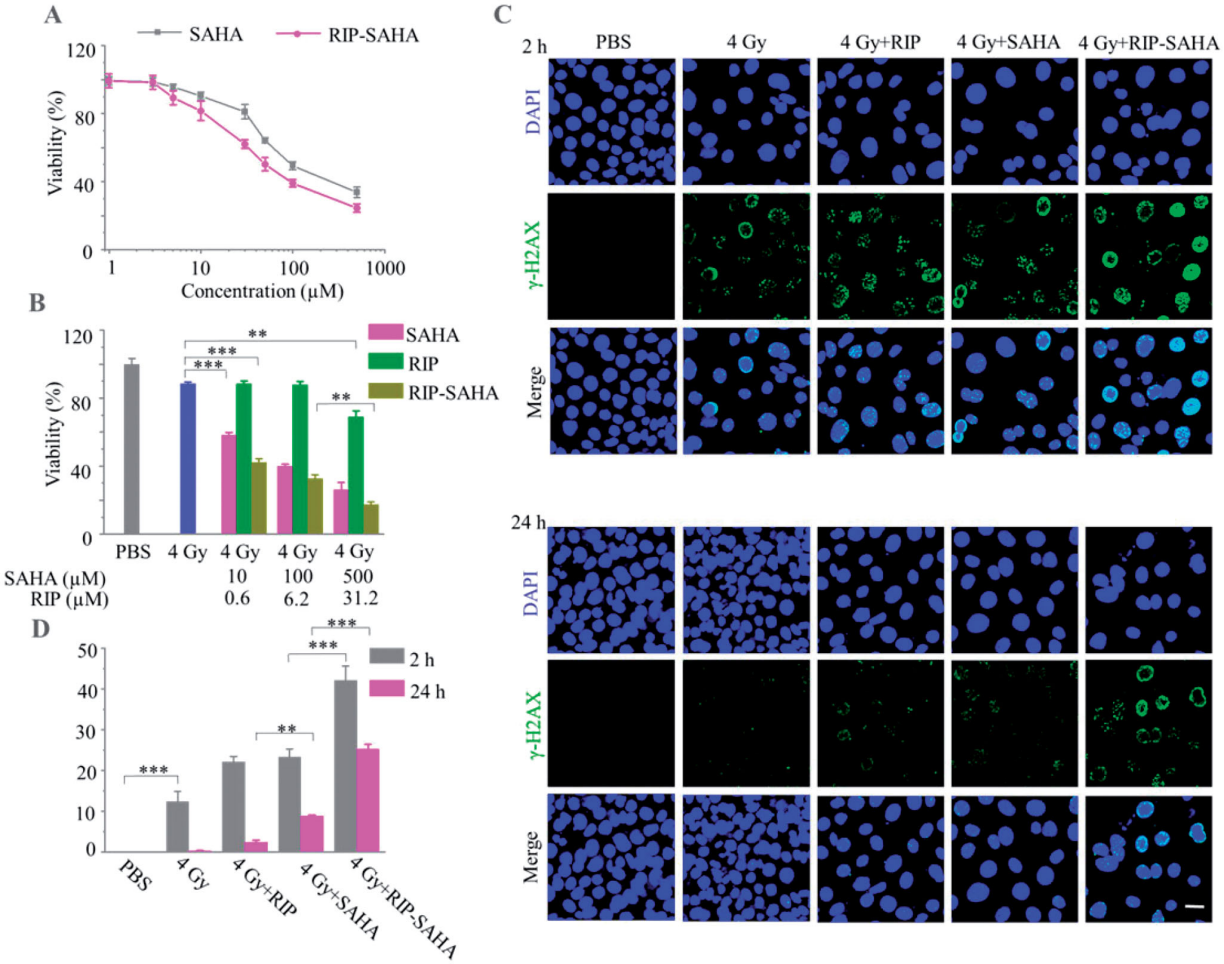
(A) MTT assays of SAHA and RIP-SAHA at SAHA concentration varying from 1 to 500 µM in 4T1 breast cancer cells following 48 h incubation. (B) MTT assays of 4T1 cells following different treatments (4 Gy of X-Ray radiation alone or plus, SAHA, RIP, or RIP-SAHA). SAHA concentration = 10, 100, and 500 μM; RIP concentration = 0.6, 6.2, and 31.2 μM. (C) Immunofluorescence assays of 4T1 cells following different treatments. The fluorescence images were scanned at 2 and 24 h after X-ray irradiation. The scale bar corresponds to 25 μm. (D) Quantification of cellular γ-H2AX foci density from fluorescence images. *p*-Values were calculated by one-way ANOVA with Tukey multiple comparison tests, **p < .01, ***p < .001.

**Figure 3.**
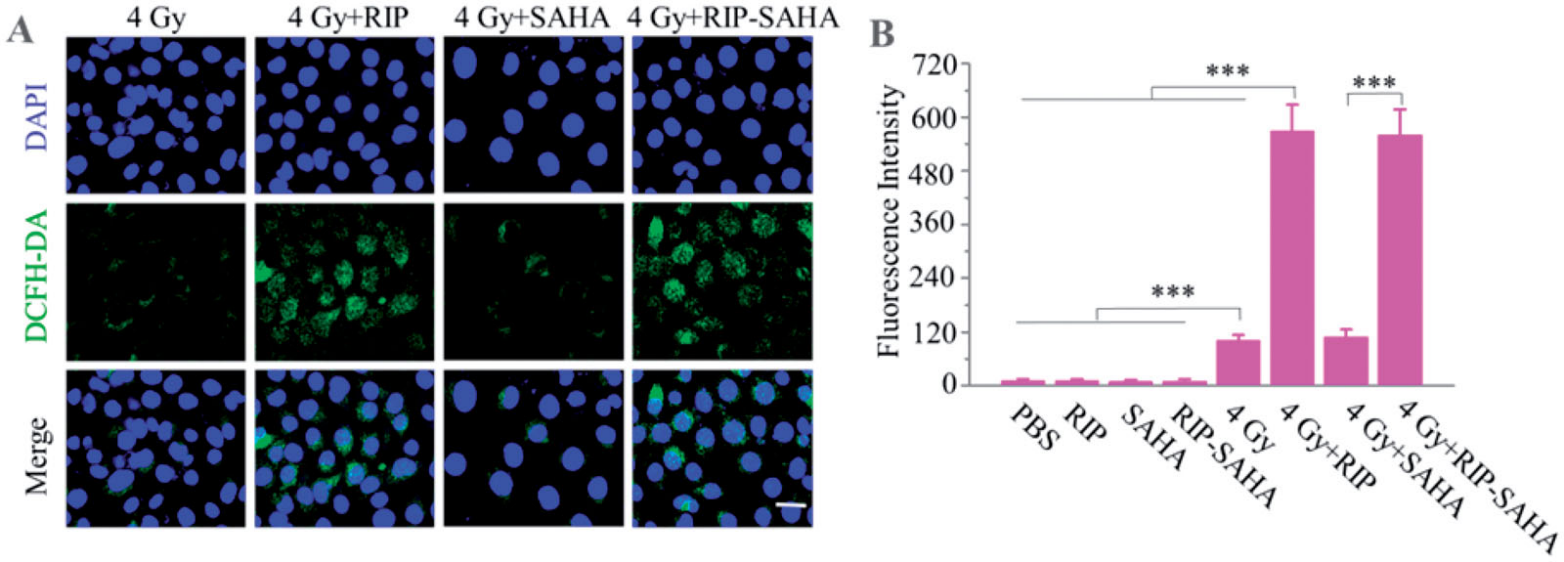
(A) Fluorescence images of ROS production in 4T1 cells after different treatment by using DCFH-DA probe. (B) Quantification of ROS production from fluorescence images. The scale bar corresponds to 25 μm. *p*-Values were calculated by one-way ANOVA with Tukey multiple comparison tests, ***p < .001.

The release studies using ^125^I-SAHA as a model drug monitored with gamma counter showed that SAHA release in 72 h increased from 33.7 ± 0.3% under the physiological condition to 64.9 ± 1.2% under 10 mM DTT condition (Figure 1(D)), confirming the reduction-triggered release of SAHA from RIP-SAHA. 4 Gy X-ray radiation, however, didn’t cause change on SAHA release from RIP-SAHA.

### Cytotoxicity

The cytotoxicity of RIP, SAHA, and RIP-SAHA was studied by methyl thiazolyl tetrazolium (MTT) assays in murine 4T1 breast cancer cells. The results showed that RIP had no obvious cytotoxicity even at a concentration of 53.3 μM (Figure S4). As expected, the viability of 4T1 cells decreased with increasing the concentration of SAHA and RIP-SAHA, in which a half-maximal inhibitory concentration (IC_50_) of 51.3 and 30.6 μM was observed for SAHA and RIP-SAHA, respectively (Figure 2(A)). The lower IC_50_ of RIP-SAHA over SAHA indicates its improved intracellular delivery.

**Figure 4.**
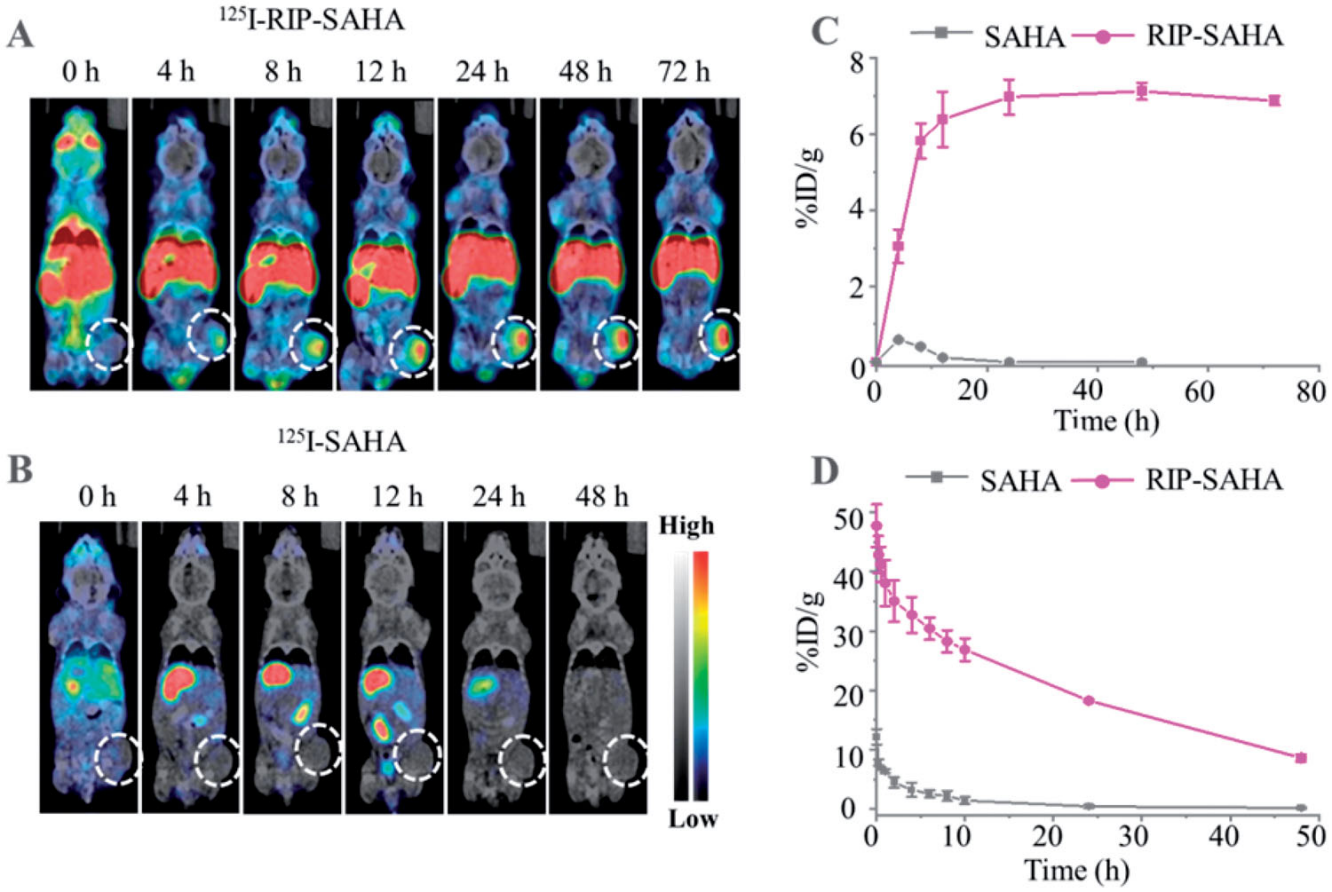
*Micro*SPECT/CT images of ^125^I-RIP-SAHA (A) and ^125^I-SAHA (200 µCi) (B) in 4T1 tumor-bearing BALB/c mice after intravenous injection over time. (C) Tumor uptake of ^125^I-SAHA and ^125^I-RIP-SAHA at different times quantified by *micro*SPECT/CT images. (D) Pharmacokinetics of ^125^I-SAHA and ^125^I-RIP-SAHA (20 µCi) in health mice following intravenous injection (*n* = 3).

SAHA is known to sensitize cancer cells toward radiotherapy. We further investigated the cytotoxicity of RIP-SAHA in combination with X-ray radiation. Figure 2(B) shows that 4T1 cells following 4 Gy X-ray irradiation had a viability of 88.5 ± 1.1%. The cell viability decreased to 58.2 ± 1.6 and 42 ± 2.3% when cells were incubated with 10 μM equiv. SAHA and RIP-SAHA, respectively, for 24 h before 4 Gy X-ray irradiation. The viability of 4T1 cells further decreased to 32.5 ± 2.4 and 17.4 ± 1.7% by increasing RIP-SAHA concentrations to 100 and 500 μM, respectively. It should further be noted that blank RIP caused a significant radiosensitizing effect at a RIP concentration of 31.2 μM, which supports that high atom number element, like iodine, can act as a radiosensitizer by generating electrons (Sarbadhikary & Dube, 2017; Cheng et al., 2018; Zhang et al., 2018), and inducing the formation of a large number of free radicals, which can kill tumor cells (Xie et al., 2019). The combination of iodine-rich nanoparticles and SAHA could synergistically enhance the DNA strain break and impede the DNA repair, thereby effectively killing tumor cells.

The immunofluorescence assay was used to investigate the DNA damage and repair of 4T1 cancer cells at 4 Gy of radiation dose. Figure 2(C) displays that X-ray radiation caused obvious DNA damage and combining 4 Gy with RIP-SAHA induced markedly more DNA damage than that with SAHA. The quantification of immunofluorescence revealed that DNA strand break foci density per cell increased significantly from X-ray treatment, X-ray combined with RIP or SAHA, to X-ray combined with RIP-SAHA (Figure 2(D)). It is interesting to note that RIP had a similar effect to SAHA at 2 h. RIP-SAHA caused not only higher foci density per cell but also a more durable effect, indicating more severe DNA damage (Nowsheen et al., 2018; Ma et al., 2019). These results indicate that RIP-SAHA can serve as a dual sensitizer to enhance X-ray radiation therapy of 4T1 cancer cells.

### Detection of reactive oxygen species (ROS)

Radiation can directly or indirectly induce DNA damage by free radical and reactive oxygen species (ROS). The production of ROS could influence the viability of tumor cells during radiotherapy. We used immunofluorescence experiments to study the production of ROS following treatment with SAHA, RIP, and RIP-SAHA with or without radiation. Without radiation, no fluorescence signal was observed in the images of SAHA, RIP, and RIP-SAHA treatments. Significant ROS signal was found in the groups of RIP or RIP-SAHA combined with X-ray radiation (Figure 3(A) and Figure S5). The quantification of immunofluorescence intensity showed that RIP or RIP-SAHA combined with X-ray radiation caused a significantly higher level of ROS than the other groups (Figure 3(B)). Free SAHA had little effect on the production of ROS.

**Figure 5.**
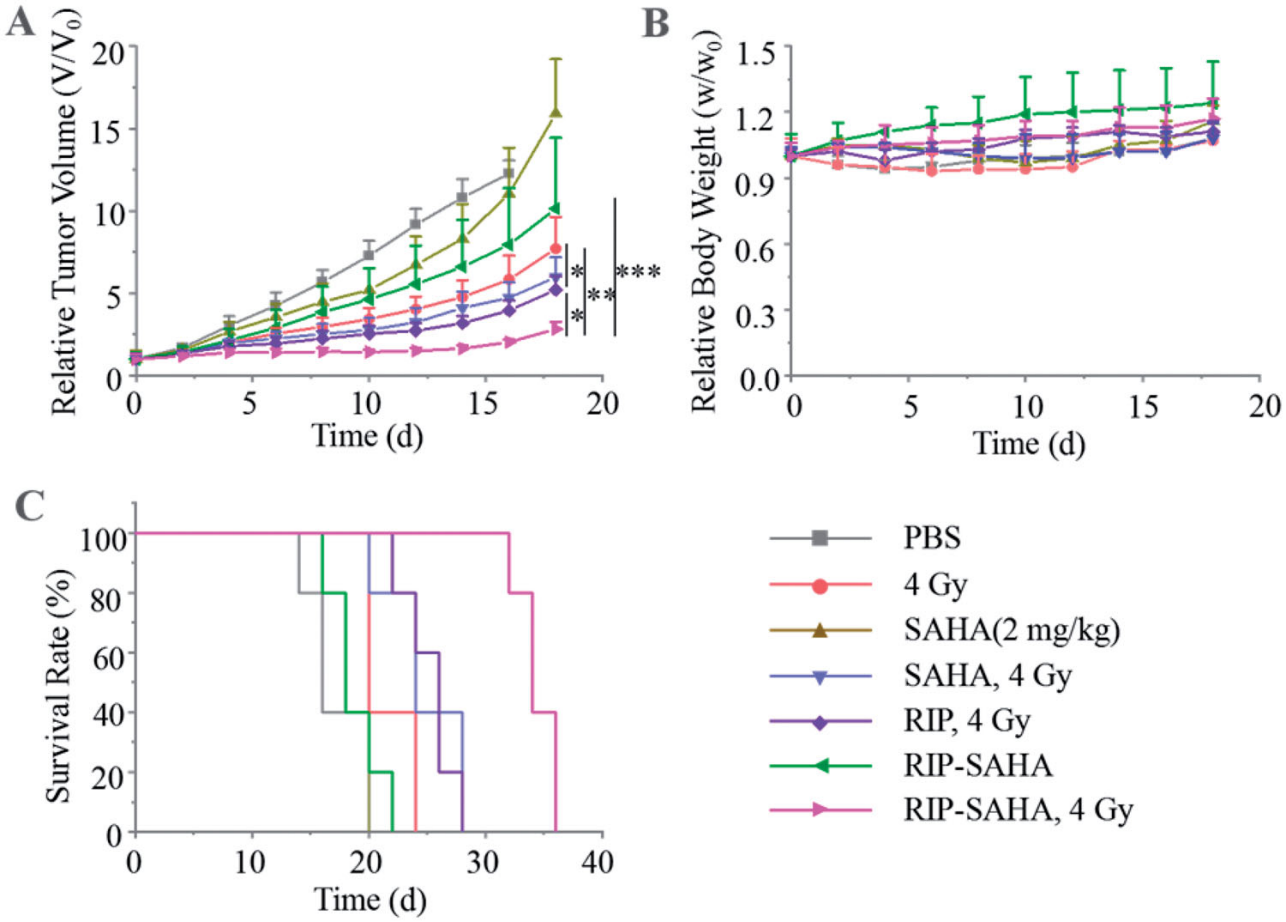
*In vivo* antitumor performance of RIP-SAHA + 4 Gy of X-ray irradiation in 4T1 tumor-bearing mice. RIP-SAHA was given on days 0, 3, and 6 and 4 Gy of X-ray irradiation was given on days 2, 5, and 8. (A) 4T1 tumor growth rate. (B) Bodyweight changes of mice. (C) Survival curves of mice (*n* = 5). *p*-Values were calculated by one-way ANOVA with Tukey multiple comparison tests, *p < .05, **p < .01, ***p < .001.

### Pharmacokinetics and biodistribution study

We further investigated the tumor accumulation of RIP-SAHA in murine 4T1 breast tumor-bearing BALB/C mice. ^125^I-RIP and ^125^I-SAHA were intravenously injected into tumor-bearing mice and imaged by *micro*SPECT. Figure 4(A) displays that ^125^I-RIP efficiently accumulated in the tumor in 8–72 h while no tumor accumulation was observed for ^125^I-SAHA (Figure 4(B)). The quantification of the *micro*SPECT images showed that tumor uptake of ^125^I-RIP increased with post-injection time and reached a plateau of 7.13 ± 0.22%ID/g at 48 post-injection. In contrast, <1%ID/g accumulation of ^125^I-SAHA was found in the tumor (Figure 4(C)). These results demonstrated that RIP could significantly increase the tumor uptake of SAHA, likely *via* the enhanced permeation retention effect (Chen et al., 2020; Zu et al., 2020). After scanning of *micro*SPECT, organs and tissues were harvested and radioactivity was measured by a gamma counter. The results showed that ^125^I-RIP mainly accumulated in the liver (24.21 ± 2.14%ID/g), spleen (17.2 ± 2.0%ID/g), and tumor (7.8 ± 0.27%ID/g) at 72 h post-injection. In contrast, <1.5%ID/g of ^125^I-SAHA was found in organs at 48 h post-injection, in line with fast excretion of SAHA from mouse body (Figure S6).

**Figure 6.**
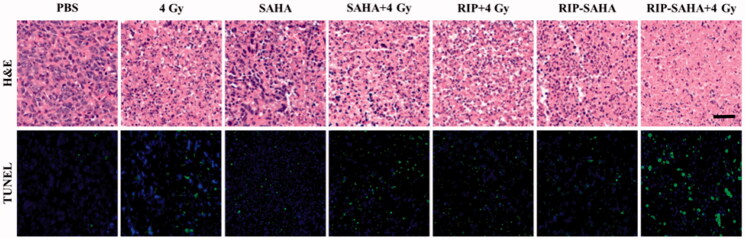
Microscopic images of H&E and TUNNEL stained tumors excised on day 11. The scale bar corresponds to 50 μm.

To study the pharmacokinetics of SAHA and RIP, ^125^I-RIP (200 μCi) and ^125^I-SAHA (200 μCi) were intravenously injected into BALB/C mice. A blood sample was drawn from the retinal vein at different post-injection times for radioactivity assay by the gamma counter. The results showed ^125^I-RIP followed a two-compartment model, in which a long blood circulation with biodistribution half-life of 0.41 h and elimination half-half of 20.38 h was observed. In contrast, ^125^I-SAHA revealed a rapidly decreased plasma level with an elimination half-life of 1.2 h. The area under the curve of ^125^I-RIP was 17.5-fold higher than that of ^125^I-SAHA (Figure 4(D)).

### In vivo antitumor therapy

Next, *in vivo* chemoradiotherapy was carried out in 4T1 breast tumor-bearing mice. The 4T1 tumor-bearing mice were randomly divided into seven groups: PBS, 4 Gy radiation, SAHA (2 mg/kg), SAHA + 4 Gy radiation, RIP + 4 Gy, RIP-SAHA, and RIP-SAHA + 4 Gy radiation. PBS, SAHA, RIP, and RIP-SAHA were intravenously injected into mice *via* tail vein at a dose of 2 mg/kg SAHA on days 0, 3, and 6. According to the biodistribution results from *micro*SPECT, 4 Gy of irradiation was given on days 2, 5, and 8. The tumor volume of mice was measured by vernier caliper and the bodyweight of mice was weighed every 2 days. RIP-SAHA seemed somewhat more effective, though not significant, than free SAHA in repressing tumor growth (Figure 5(A)). Notably, the combination of RIP-SAHA with 4 Gy X-ray radiation led to potent inhibition of 4T1 tumors, which was significantly more effective than SAHA + 4 Gy radiation, RIP + 4 Gy radiation, or 4 Gy radiation alone. It should further be noted that both RIP and SAHA improved tumor radiotherapy with 4 Gy radiation, supporting that both RIP and SAHA are effective radiosensitizers. Importantly, no obvious body weight loss was found for all groups (Figure 5(B)), indicating that radiosensitizer-enhanced radiotherapy is a low toxic strategy to improve tumor therapy. The Kaplan-Meier survival curve displayed that RIP-SAHA + 4 Gy irradiation group had a significantly improved survival rate compared with all the other groups (Figure 5(C)). SAHA or RIP combined with 4 Gy irradiation showed only modest survival benefits over X-ray irradiation alone. The hematoxylin-eosin (H&E) and TUNEL staining of tumor slices displayed that RIP-SAHA + 4 Gy radiation caused more serious necrosis and apoptosis of 4T1 cells than all other treatment groups (Figure 6). The H&E images of normal organs didn’t show obvious damage (Figure S7). The above results indicate that RIP-SAHA + 4 Gy irradiation can effectively kill tumor cells without damaging normal tissues.

## Conclusion

We have demonstrated that suberoylanilide hydroxamic acid (SAHA)-loaded redox-sensitive iodinated polymersomes (RIP-SAHA) are novel and smart dual-functional nano-radiosensitizer that significantly enhances tumor radiotherapy without causing additional toxic effects. The disulfide-crosslinking and nano-sized feature of RIP-SAHA has not only greatly improved its stability, pharmacokinetics, and tumor accumulation but also facilitated the intracellular release of SAHA in the tumor cells, leading to impaired DNA repair and enhanced DNA damage. The high iodine nature of RIP further enhances the sensitization of tumor cells to radiotherapy. These dual-functional nano-radiosensitizer has offered a unique platform for enhanced cancer radiotherapy.

## Supplementary Material

Supplemental Material
