# Supplementary material for: Redox-sensitive iodinated polymersomes carrying histone deacetylase inhibitor as a dual-functional nano-radiosensitizer for enhanced radiotherapy of breast cancer
Source: Drug Deliv. 2021 Nov 3;28(1):2301–9. doi: 10.1080/10717544.2021.1995080 (PMC8567935; doi:10.1080/10717544.2021.1995080)
Supplement: Supplemental Material [file IDRD_A_1995080_SM7812.docx]

**Redox-Sensitive Iodinated Polymersomes Carrying Histone Deacetylase Inhibitor as a Dual-Functional Nano-Radiosensitizer for Enhanced Radiotherapy of Breast Cancer**

Zhehong Zhu^1^, Manran Wu^2^, Juan Sun^1^, Zhengyuan Huangfu^1^, Lingling Yin^2^, Weipeng Yong^2^, Jing Sun^2^, Guanglin Wang^2,*^, Fenghua Meng^1^, Zhiyuan Zhong^1, *^

^1^ Biomedical Polymers Laboratory, College of Chemistry, Chemical Engineering and Materials Science, and State Key Laboratory of Radiation Medicine and Protection, Soochow University, Suzhou 215123, People’s Republic of China.

^2^ State Key Laboratory of Radiation Medicine and Protection, School of Radiation Medicine and Protection & School for Radiological and Interdisciplinary Sciences (RAD-X), Collaborative Innovation Center of Radiation Medicine of Jiangsu Higher Education Institutions, Soochow University, Suzhou 215123, People’s Republic of China.

**Corresponding author:**

E-mail: [glwang@suda.edu.cn](mailto:glwang@suda.edu.cn); Tel: +86-512-65880054

E-mail: [zyzhong@suda.edu.cn](mailto:zyzhong@suda.edu.cn); Tel: +86-512-65880098


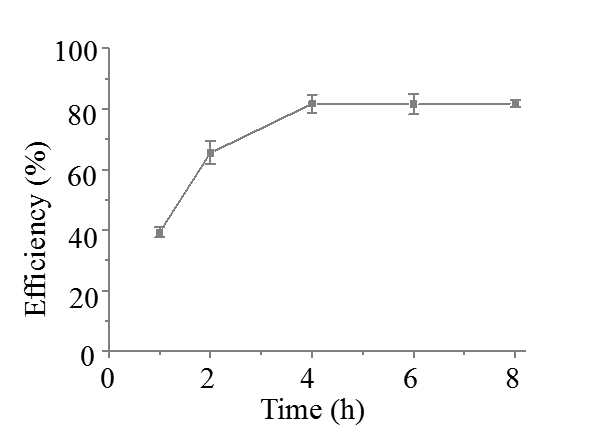


**Figure S1.** Radiolabeling efficiency of ^125^I-RIP of reaction time measured by TLC.


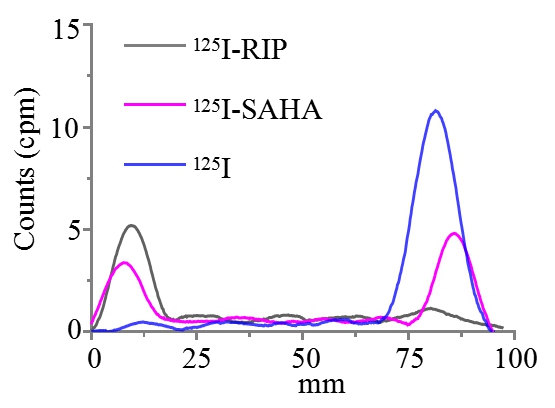


**Figure S2.** The Rf of ^125^I, ^125^I-SAHA, and ^125^I-RIP using saline as a mobile phase.


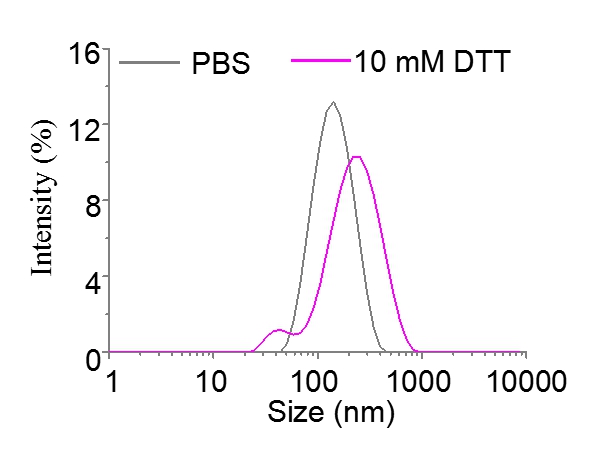


**Figure S3.** Hydrodynamic size and biodistribution of RIP with PBS (pH 7.4) or 10 mM DTT measured by DLS.


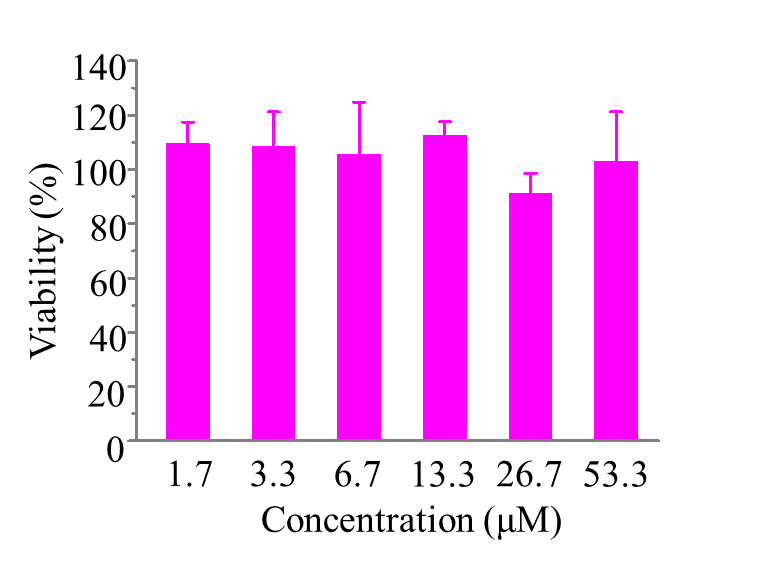


**Figure S4.** MTT assays of RIP with concentration varying from 1.7 μM to 53.3 μM.


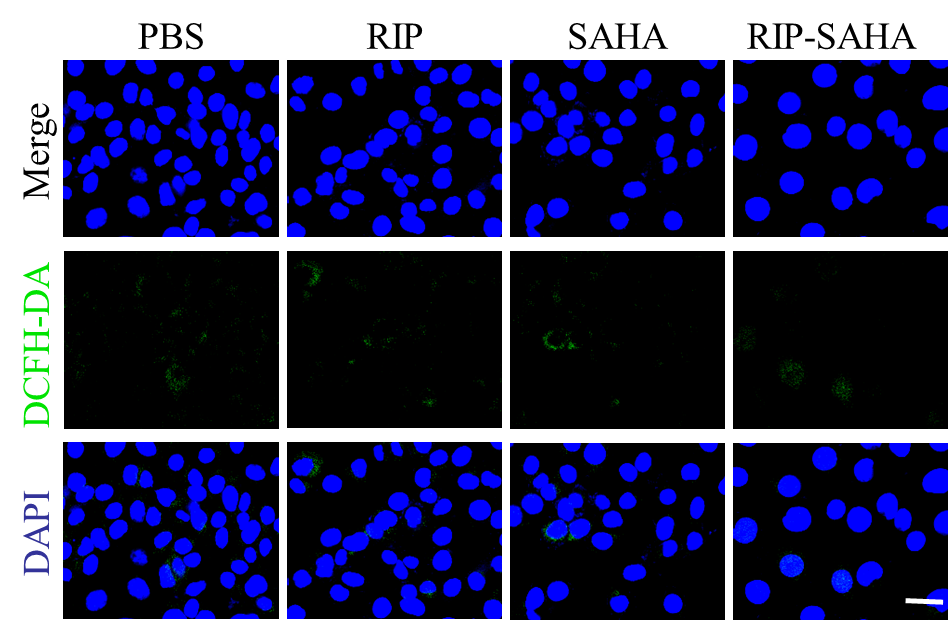


**Figure S5.** Fluorescence images of ROS production in 4T1 cells with different treatment by using DCFH-DA probe.


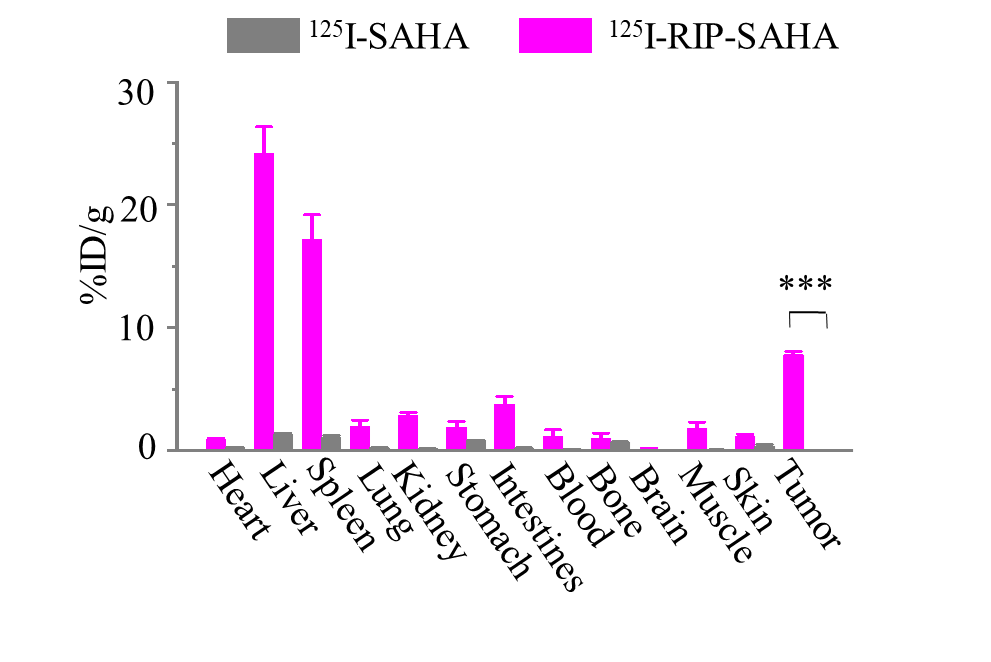


**Figure S6.** Biodistribution of ^125^I-SAHA and ^125^I-RIP-SAHA (200 µCi) in 4T1 tumor-bearing BALB/c mice after intravenous injection at 48 h and 72 h, respectively (n=3). P values were calculated by One-way ANOVA with Tukey multiple comparison tests, p*** <0.001.


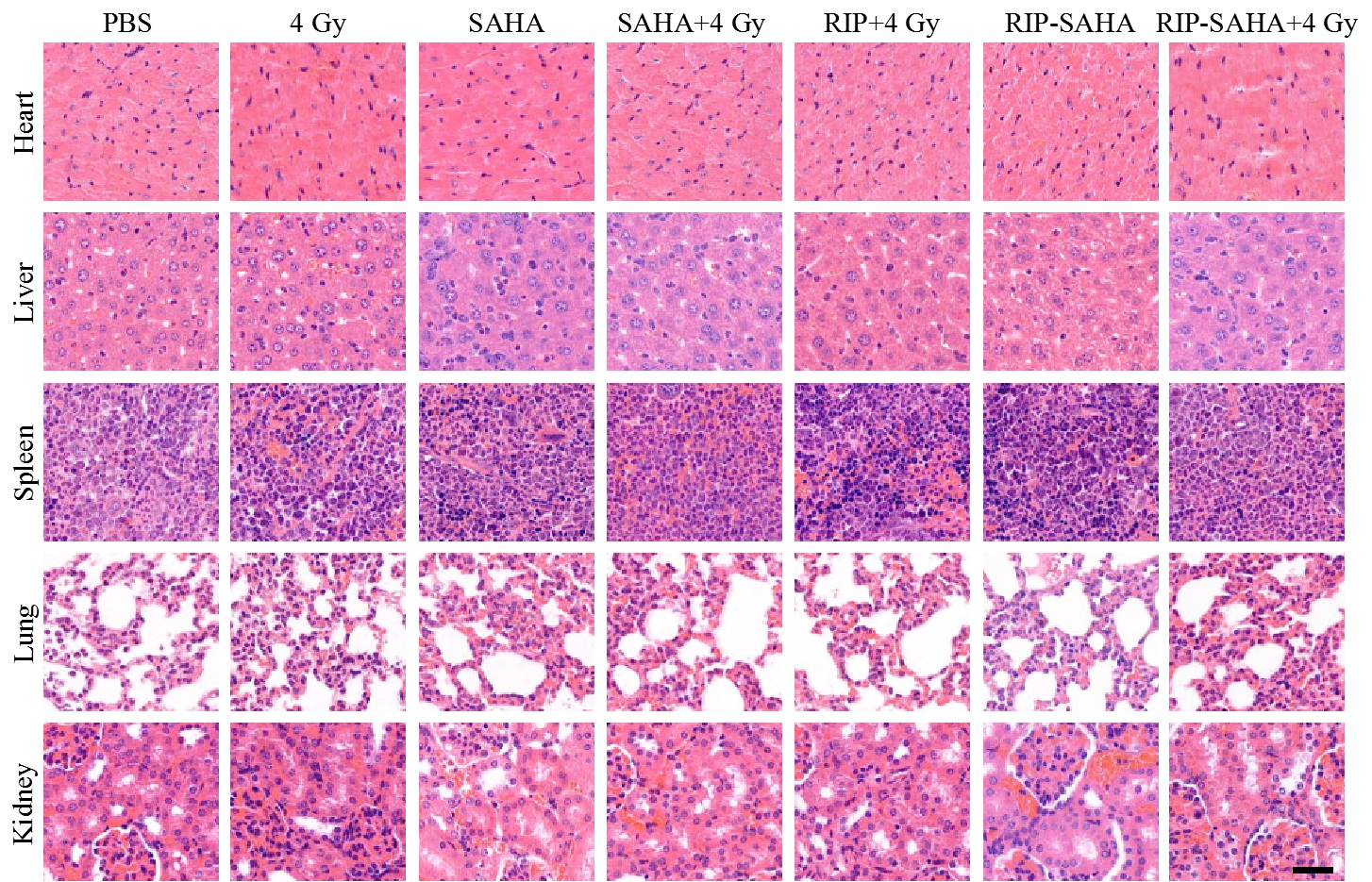


**Figure S7.** Microscopic images of H&E stained of heart, liver, spleen, lung, and kidney excised on day 11. The scale bar corresponds to 50 μm.
